# Making High Thermoelectric and Superior Mechanical Performance Nb_0.88_Hf_0.12_FeSb Half‐Heusler via Additive Manufacturing

**DOI:** 10.1002/advs.202403705

**Published:** 2024-09-09

**Authors:** Zhifu Yao, Wenbin Qiu, Chen Chen, Xin Bao, Kaiyi Luo, Yong Deng, Wenhua Xue, Xiaofang Li, Qiujun Hu, Junbiao Guo, Lei Yang, Wenyu Hu, Xiaoyi Wang, Xingjun Liu, Qian Zhang, Katsumi Tanigaki, Jun Tang

**Affiliations:** ^1^ Department of Fundamental Courses Wuxi Institute of Technology WuXi 214121 China; ^2^ School of Materials Science and Engineering and Institute of Materials Genome & Big Data Harbin Institute of Technology Shenzhen 518055 China; ^3^ Key Laboratory of Radiation Physics and Technology of Ministry of Education Institute of Nuclear Science and Technology Sichuan University Chengdu 610064 China; ^4^ State Ethnic Affairs Commission Southwest Minzu University Chengdu 610041 China; ^5^ College of Physics Sichuan University Chengdu 610064 China; ^6^ School of Materials Science & Engineering Sichuan University Chengdu 610064 China; ^7^ Materials Characterization and Preparation Center and Department of Physics Southern University of Science and Technology Shenzhen 518056 China; ^8^ Division of Quantum State of Matter Beijing Academy of Quantum Information Sciences Beijing 100193 China

**Keywords:** additive manufacturing, half‐Heusler, laser powder bed fusion, thermoelectric

## Abstract

Thermoelectric generators held great promise through energy harvesting from waste heat. Their practical application, however, is greatly constrained by poor raw material utilization and tedious processing in fabricating desired shapes. Herein, a state‐of‐the‐art process is reported for 3D printing the half‐Heusler (Nb_0.88_Hf_0.12_FeSb) thermoelectric material using laser powder bed fusion (LPBF). The multi‐dimensional intra‐ and inter‐granular defects created by this process greatly suppress thermal conductivity by providing numerous phonon scattering centers. The resulting LPBF‐fabricated half‐Heusler exhibits a high figure of merit ≈1.2 at 923 K and a single‐leg maximum efficiency of ≈3.3% at a temperature difference (*ΔT*) of 371 K. Hafnium oxide nanoparticles generated during LPBF effectively prevent crack propagation, ensuring competent mechanical performance and reliable thermoelectric output. The findings highlight the significant potential of LPBF in driving the next industrial revolution of highly efficient and customizable thermoelectric materials.

## Graphical abstract

Nb_0.88_Hf_0.12_FeSb, a half‐Heusler thermoelectric material for energy harvesting, exhibits a very promising thermoelectric figure of merit *ZT* ≈ 100 °C. With the application of laser‐based 3D printing technique onto high‐quality spherical precursor powder, highly flexible shape‐customization, and efficient prolificacy can be further achieved with negligible degeneration in thermoelectric and mechanical performances.

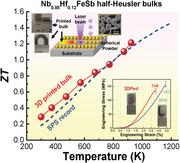

## 1 Introduction

In light of the impending energy crisis, the concept of carbon neutrality is put forward and a sustainable electricity supply is essential for alleviating the burden on the global environment. Thermoelectric materials are capable of converting waste heat into electricity or vice versa with the performance determined by the dimensionless figure of merit *ZT* = (*S* ^2^ *σ*/*κ*_tot_). *T*, where *S*, *σ*, *κ*_tot_, and *T* refer to the Seebeck coefficient, the electrical conductivity, the total thermal conductivity, and the temperature, respectively.^[^ ^1^ ^]^ Half‐Heusler (HH) based thermoelectric generators (TEG) are a promising option for heat harvesting at temperatures of hundreds of degrees Celsius.^[^ ^2^ ^]^ Whereas, their labor‐intensive fabrication process^[^ ^3^ ^]^ poses significant challenges to widespread applications. Despite of the continuous advances in materials science,^[^ ^4^ ^]^ the rigidity of the HH family^[^ ^5^ ^]^ always remains a hindrance to efficient fabrication methods. Thus, innovative fabrication techniques with higher production efficiency and flexibility are hitherto imperative to overcome the bottleneck in manufacturing high‐performing HH‐TEG_s_.^[^ ^6^ ^]^

Compared to conventional manufacturing involving complex procedures, additive manufacturing (also known as 3D printing, 3DP) is an attractive digital shaping technique featuring by discrete‐collecting principle.^[^ ^3^, ^7^ ^]^ It offers advantages in manufacturing efficiency and waste reduction by enabling the production of complex geometries, minimizing material waste, allowing for on‐demand production, and simplifying assembly processes. Although various studies ≈ of 3DP‐prepared thermoelectric materials were reported, they mostly focused on Bi_2_Te_3_ using liquid‐based precursors.^[^ ^8^ ^]^ Laser powder bed fusion (LPBF) is the present mainstream among 3DP methods for large‐scale^[^ ^9^ ^]^ near‐net‐shape forming. Significant material waste and shape limitations in current manufacturing are expected to be no longer problematic. Moreover, the ultrahigh‐energy input and rapid solidification rate of LPBF methods can realize the complete synthesis of materials with high melting points and simultaneously ensure excellent mechanical strength, which prevails over the preparation methods involving organic mediums. Some attempts of LPBF were demonstrated for Bi_2_Te_3_,^[^ ^8^, ^10^ ^]^ skutterudites,^[^ ^11^ ^]^ and SnTe^[^ ^12^ ^]^ in recent years. Nevertheless, one has been encountering extra serious difficulties in the LPBF process for HH caused by the sharp difference in melting points,^[^ ^13^ ^]^ and thermal stress accumulation,^[^ ^14^ ^]^ and poor wettability.^[^ ^15^ ^]^ Although demonstrations of LPBF‐deposited HH were shown for n‐type ZrNiSn^[^ ^14^, ^16^ ^]^ and p‐type Hf_0.3_Zr_0.7_CoSn_0.3_Sb_0.7_^[^ ^16^ ^]^ in the past, the poor flowability of powders commercially available significantly has been limiting both manufacturing efficiency and scale‐up prospect. Consequently, well‐regulated stacking and uniformity are warranted in order to solve the aforementioned problems in the case of LPBF‐HH.

For the ideal utility of the LPBF^[^ ^17^ ^]^ process, gas atomization is preferred to produce spherical‐shaped powders from equiaxial ones. To our knowledge, the successful attempts of spherical‐shaped powders as a prerequisite for LPBF in thermoelectric materials were very limited^[^ ^18^ ^]^ and none was reported for the HH family. Further investigation into the powder atomization followed by LPBF printing is required to validate the simplicity and effectiveness of this process in a variety of materials.^[^ ^6^ ^]^ LPBF offers substantial flexibility in customizing the geometry of thermoelectric devices, making it ideal for designing and fabricating novel device architectures.^[^ ^19^ ^]^ In this study, we demonstrate a successful integration of LPBF to fabricate Nb_0.88_Hf_0.12_FeSb,^[^ ^2^, ^20^ ^]^ a heavy‐band HH compound with great potential as a low‐cost, high‐performance mid‐to‐high‐temperature power‐generation module with good thermal and mechanical stability. The spherical‐shaped powders were prepared by gas atomization. Our results show that both high thermoelectric performance and shape customization can be achieved through LPBF. These findings will enlighten an innovative manufacturing process leading to practical thermoelectric applications by simply eliminating the demands for separate components and dramatically reducing assembly steps.

## 2 Results and Discussion

### 2.1 Processing and Basic Properties of Products

**Figure 1:**
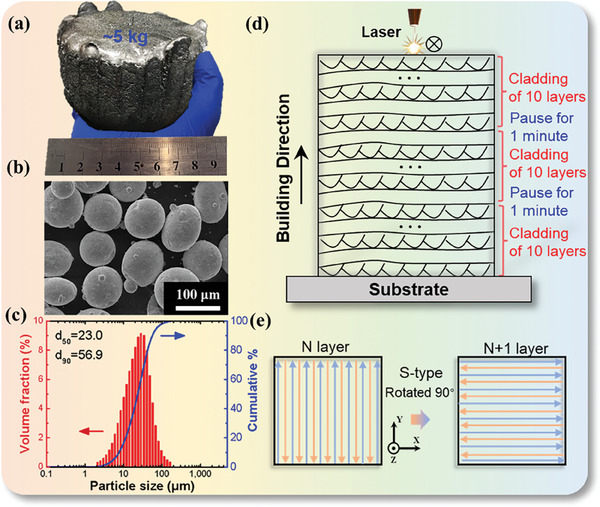
The processing route of the LPBF‐based 3D printing for half‐Heusler in this work. a) Photograph of a cast ingot after levitation melting (LM), inset showing the appearance of melt during LM process. b) SEM secondary electron image of spherical powder. c) Particle size distribution of spherical powder. d) A schematic of the LPBF process (after cladding 3 layers in a sequence, the process was paused for 1 min, to avoid over‐heat accumulation). e) Laser scanning strategy within adjacent printing layers.

**Figure** **1** illustrates the LPBF‐based 3DP technology employed in this work. Figure 1a illustrates the kilogram‐scale ingot of Nb_0.88_Hf_0.12_FeSb obtained through levitation melting (LM). The morphology of the spherical Nb_0.88_Hf_0.12_FeSb powder atomized from ingots is illustrated in Figure 1b. The corresponding cross‐sectional SEM image (Figure S1, Supporting Information) confirms the spherical shape and hollow‐free property. Figure 1c provides the particle size dependence of volume fraction and cumulative percentage. The percentile values d_50_ and d_90_ of spherical powder are 23.0 µm and 56.9 µm, respectively, to meet the standard LPBF requirements.^[^ ^21^ ^]^ Specifically, to mitigate the accumulation of heat resulting from the inherently low thermal conductivity of thermoelectric materials, a 1‐min pause is implemented after the melting of every ten layers during the printing process. This interlude ensures an adequate cooling period (Figure 1d). Additionally, the laser scanning strategy within each layer and the adjacent printing layers are shown in Figure 1e.

**Figure 2:**
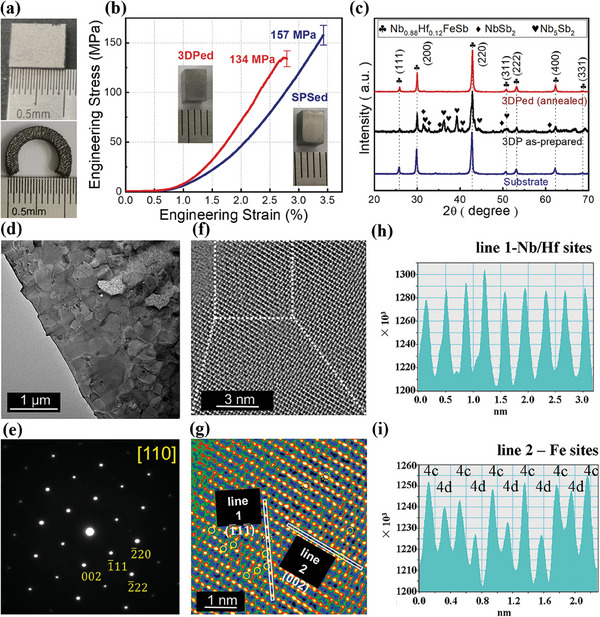
Some basic properties of the ingot, spherical powder, and LPBF Nb_0.88_Hf_0.12_FeSb bulk products were obtained in this work. a) Photographs of the LPBF‐prepared cuboid and arc shape samples. b) Compressive stress‐strain curves of SPS bulk and 3DPed samples with the corresponding photographs shown in the insets. c) XRD 2θ scanning for printing substrate, 3DP as‐prepared and 3DP annealed (named “3DPed”) samples. d,e) Low‐mag image and the corresponding SAED patterns for HH parent phase. f) High‐angle annular dark field‐scanning transmission electron microscopy (HAADF‐STEM) image. g) A colored HAADF‐STEM image displaying the magnified region selected in (f). The alignments of constituent atoms are clearly distinguished. Two profiles, line 1 (h) and line 2 (i), are conducted.

The appearance, crystal structure, and mechanical properties of the products obtained from all steps described earlier are provided in **Figure** **2**. The LPBF‐printed samples exhibit a relatively high mass density ≈8.68 g cm^−3^ (over 92% of theoretical value), demonstrating the capability of LPBF in preparing HH thermoelectric bulks. The appearance of an LPBF‐prepared Nb_0.88_Hf_0.12_FeSb cuboid is shown in Figure 2a. Shape‐conformability is one of the most important advantages of 3DP,^[^ ^22^ ^]^ which enables a fitting shape to an established surface regardless of geometric complexity.^[^ ^6^ ^]^ Figure 2a also exhibits an arc‐shaped HH object that is ideal for waste heat harvesting on circular water pipes,^[^ ^8a^ ^]^ manifesting the flexibility of LPBF in fabricating irregular geometries. Figure 2b shows the compressive strengths of 134, and 157 MPa for 3DPed and SPSed bulks, respectively. Additionally, the hardness values of the 3DPed and SPS samples are 72.3 ± 8.1 and 76.3 ± 2.4 HRC, respectively, with no significant difference (Figure S2, Supporting Information). The LPBF‐prepared HH bulk exhibits a comparable mechanical performance as the conventionally prepared one, reflecting the potential of LPBF in fabricating robust HH‐based TEGs. X‐ray diffraction (XRD) patterns of the substrate, 3DP as‐prepared (without post‐annealing) and 3DPed (annealed), are shown in Figure 2c. The results show a miscellaneous composition for the 3DP as‐prepared sample and it transforms to a pure HH phase after the introduction of post annealing which prompts the decomposition and integration of metastable secondary phases. Based on energy dispersive X‐ray spectrum (EDS) results (Figure S3, Supporting Information), the chemical composition of the 3DPed bulk is Nb: Hf: Fe: Sb ≈ 30.1: 3.8: 33.2: 32.9, which is very close to the nominal stoichiometry (0.88: 0.12: 1: 1). For both the 3DPed and SPS samples, they exhibit a near‐equiaxed grain morphology, with average grain sizes of 304 ± 142 and 331 ± 79 nm, respectively, indicating minimal difference (Figure S4, Supporting Information). Besides, the grain size distributions are shown in Figure S5 (Supporting Information).

The microstructure of 3DPed is comprehensively characterized by TEM as shown in Figure 2d,g. The low‐mag TEM image and corresponding diffraction and fast Fourier transform (FFT) patterns in Figure 2d,e verify the domination of the Nb0.88Hf0.12FeSb phase in the matrix. The occasional occupancy of iron atoms at the 4d sites in the HH structure (Figure 2f,i) suggests the possible formation of full‐Heusler (FH) phases. Nb/Hf sites and Fe sites are respectively marked by dashed and solid circles. The observed intensity fluctuation indicates the fact that Fe atoms occasionally occupy the 4d sites in addition to regular 4c sites in the 3DPed sample. The excess occupancy is quite common in both p‐type^[^ ^2a^ ^]^ and n‐type^[^ ^23^ ^]^ HH compounds containing nanosized full‐Heusler (FH) inclusions, which can improve thermoelectric performances by dramatically suppressing lattice thermal conductivity *κ*_lat_^[^ ^23^, ^24^ ^]^ (“lat” is short for “lattice”).

### 2.2 Electrical and thermal transport properties

**Figure 3:**
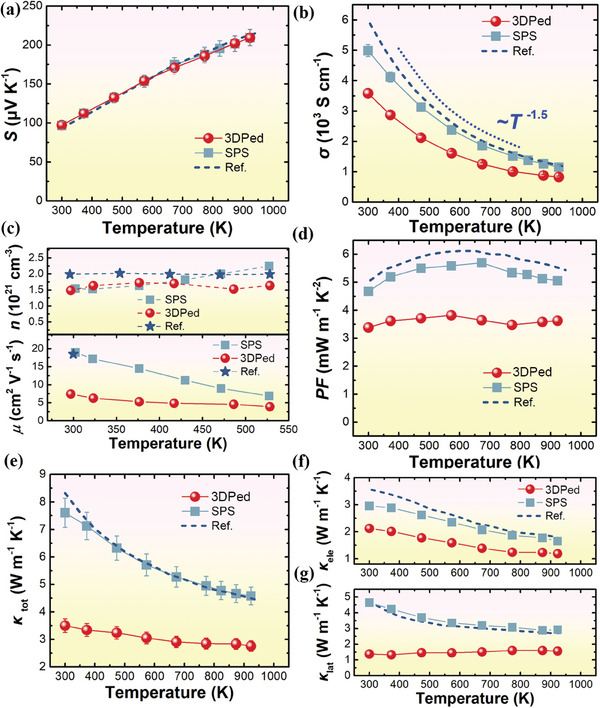
Temperature dependence of electrical and thermal transport properties of 3DPed, as‐grown SPS, and the reference. a) Seebeck coefficient *S*. b) electrical conductivity *σ*. c) carrier mobility *µ* and carrier concentration *n*. d) power factor *PF*. e) total thermal conductivity *κ*_tot_. f) electron thermal conductivity *κ*_ele_. g) lattice thermal conductivity *κ*_lat_. The performances in the reference^[^ ^2a^ ^]^ are plotted for comparison.

Thermoelectric properties of the 3DPed sample were parallelly investigated with the SPSed one and the Nb_0.88_Hf_0.12_FeSb bulk published for the first time^[^ ^2a^ ^]^ (denoted as “ref.” in the rest of the article). SPS, the common powder metallurgy method for thermoelectric preparation, was used to fabricate both SPSed and ref. samples with identical parameters. The electrical transport properties, including the temperature dependences of *S*, *σ*, carrier mobility (*µ*), carrier concentration (*n*), and the power factor (*PF* = *S* ^2^ *σ*), are provided in **Figure** **3**. As shown in Figure 3a,b, both *S*‐*T* and *σ*‐*T* relationships of SPSed were found to be similar to those of the reference,^[^ ^2a^ ^]^ indicating the high quality of the spherical powder without obvious deterioration after the vacuum induction melting inert gas atomization (VIGA) process. The *σ* of 3DPed is ≈25% lower than that of the SPS sintered sample, which is mostly attributed to the deteriorated electron transport caused by the non‐fully dense nature and the multiple types of defects generated in the LPBF‐prepared sample as to be discussed later. The temperature dependence of *T* ^−1.5^ implies the dominant contributions of acoustic phonon‐scatterings in electrical transport. The Hall measurements (Figure 3c) indicate a room‐temperature *n* value of 3DPed ≈1.5 × 10^21^ cm^−3^, which is close to the value of 2 × 10^21^ cm^−3^ for Nb_0.88_Hf_0.12_FeSb with excellent thermoelectric performance.^[^ ^2a^ ^]^ Based on the equation of *σ* = *neµ* (product of carrier concentration *n*, elementary charge *e*, and carrier mobility *µ*), *µ*‐*T* relationships are calculated and exhibit a much lower level of *µ* in the LPBF‐prepared samples than the other two sintered by SPS. Hence, the *n* value in this work resides in the optimized range, guaranteeing a fairly competitive *PF* among p‐type HH composites^[^ ^25^ ^]^ in the 3DPed samples despite the suppressed *σ*, as shown in Figure 3d. The temperature dependence of *κ*_tot_ is exhibited in Figure 3e. Similar to the electrical transport results, the SPS sample also shows a similar temperature‐dependent *κ*_tot_ to that of the reference. In 3DPed samples, *κ*_tot_ remains to be low thanks to the non‐fully dense and specific microstructure of the LPBF‐prepared samples. In comparison to SPS, the 3DPed sample shows a dramatical reduction in *κ*_tot_ by over 60% in the entire temperature range.

To clarify the thermal transport via phonons, the lattice thermal conductivity *κ*_lat_ = *κ*_tot_ – *κ*_ele_ is estimated by subtracting the electronic contribution from *κ*_tot_. According to the Wiedemann‐Franz law *κ*_ele_ = *LσT*, *κ*_ele_ can be quantified by the product of *σ*, *T*, and the Lorentz number *L*, which is obtained by fitting a single parabolic band (SPB) model.^[^ ^26^ ^]^ The temperature dependence of *κ*_ele_ and *κ*_lat_ are plotted in Figure 3f,g, respectively. As the carrier concentrations are quite close between both samples, the relatively lower *κ*_ele_ in 3DPed is ascribed to the inferior mobility. The *κ*_lat_ of 3DPed is significantly suppressed down to 1.3 W m^−1^ K^−1^. Since LPBF is a nonequilibrium dynamic process, it is worth investigating the mechanisms of the particular microstructural characteristics on the surprisingly low *κ*_lat_ realized in 3DPed, which will be investigated in the next section.

### 2.3 Nanostructured Features

**Figure 4:**
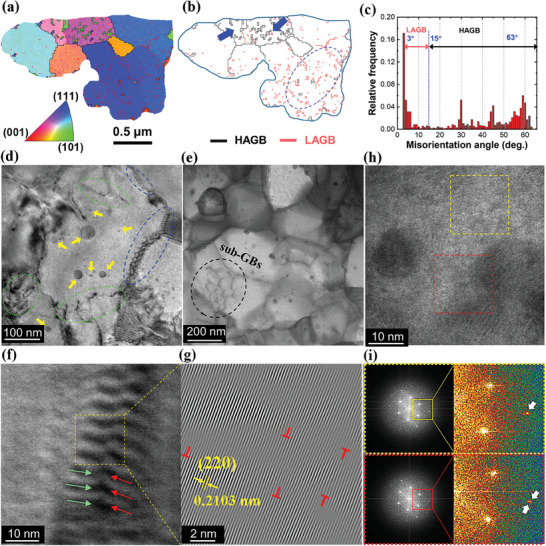
EBSD‐TKD results of the 3DPed sample observed from the plane parallel to the building direction. a) The inverse pole figure (IPF) map of a region cut along intact grain boundaries (GBs) with a step size of 0.02 µm. b) The EBSD‐TKD grain boundary (GB) map for the same region. The typical areas that are rich in low‐angle GBs (LAGB) and high‐angle GBs (HAGB) are indicated by dashed circles and arrows, respectively. c) The statistical distribution results of the misorientation angle were taken from a rectangular area with 3.0 µm in length and 1.6 µm in width. d,e) Low‐mag TEM image showing a variety of defects: nano‐precipitates (arrows), GB dislocation arrays (dashed circles), and line defects (dotted circles). f) A region with intersected GB dislocation arrays. g) Inverse fast Fourier transform (IFFT) of the square in (f) showing dislocations. h,i) HRTEM and fast Fourier transform (FFT) images demonstrating the determination of FH nano‐precipitates.

Given the extremely high cooling rate of the LPBF process (10^3^–10^8^ °C s^−1^),^[^ ^21^ ^]^ the growth of grains is effectively suppressed. Herein, the electron backscatter diffraction ‐transmission Kikuchi diffraction (EBSD‐TKD) is employed to analyze the nanosized HH grains of an LPBF bulk. The plane for observation is parallel to the building direction of LPBF. An inverse pole figure (IPF) map is illustrated in **Figure** **4**a. Note that the 3DPed sample contains no columnar grain which frequently causes anisotropy in LPBF processes.^[^ ^27^ ^]^ The corresponding grain boundary (GB) map is given in Figure 4b showing the distribution of high‐angle‐grain‐boundaries (HAGBs, misorientation angle ≥15°) and low‐angle‐grain‐boundaries (LAGBs, misorientation angle <15°). In the case of LPBF, intragranular sub‐grain‐boundaries (sub‐GBs) are usually recognized as LAGBs generated by the unique solidification process of LPBF, such as the local temperature fluctuation and the unstable melt pool.^[^ ^28^ ^]^ In addition, numerous looped HAGBs with tens of nanometers in width firmly embed inside HH grains. Given the identical crystal orientations of the nanosized HAGBs in individual HH grains, they are classified as nano‐precipitates with similar properties, which are to be discussed based on high‐resolution transmission electron microscopy (HRTEM) results later. The distribution of the misorientation angle (Figure 4c) indicates a large proportion of HAGBs. Both LAGBs and HAGBs are anticipated to impose significantly large influences on the carrier and phonon transport properties of thermoelectric compounds^[^ ^29^ ^]^ particularly near room temperature.^[^ ^30^ ^]^

By focusing on HH grains in a higher magnification, a variety of nanosized intra‐ and inter‐granular defects with multiple magnitudes of scales are observed in Figure 4d, including nano‐precipitates, GB dislocation arrays, line defects, etc. In Figure 4e, a cluster of sub‐GB networks is distinguished, which is consistent with the existence of massive LAGBs indicated by EBSD‐TKD results (Figure 4b). The positive contribution of nanosized sub‐GBs in suppressing the phonon transport has been widely demonstrated in various thermoelectric systems,^[^ ^31^ ^]^ achieved by the formation of dislocation arrays and strain clusters. Figure 4f captures a zip‐shaped feature consisting of intersected dislocation arrays piling up at a GB. Given that the possibility of elemental fluctuation is ruled out by the evidence of EDS linear scanning (Figure S6, Supporting Information), the zip feature at GB is recognized as intensive strains generated from the high‐density edge dislocations (Figure 4g), which is similar to the phenomena of Moiré patterns observed elsewhere.^[^ ^29^, ^31^ ^]^ An HRTEM image of an HH grain with nano‐precipitates is illustrated in Figure 4h. Two separate regions with and without nano‐precipitates are parallelly compared through FFT. As shown in Figure 4i, the two FFT images are mostly identical except for the additional batch of diffraction patterns emerging in the region containing nano‐precipitates (red square highlighted in Figure 4h). Considering the same space group (F‐43m), larger values of d‐spacings, and distinct Fe‐rich nature (Figure S7, Supporting Information), the nano‐precipitates in Figure 4h are therefore identified as the FH nano‐inclusions. They also exhibit HAGB loops with identical orientations in EBSD‐TKD (Figure 4b) owing to similar size and concentration inside HH grains. The introduction of FH nano‐precipitates can cause dramatic strain‐induced lattice distortions in HH composites.^[^ ^32^ ^]^ Based on the previous reports, diverse dislocations have been observed in many 3DP‐prepared metals.^[^ ^33^ ^]^ As for the physical origin of dislocations in LPBF‐prepared counterparts, Wang et. al developed multi‐physics modeling^[^ ^33c^ ^]^ and demonstrated that repeated compression‐tension cycles rendered by the local thermal inhomogeneity gave a major contribution to the formation of high‐density dislocation. It suggests that the unique process of LPBF is the origin of high‐density dislocations rather than metallic interactions, which is also explicable to the multi‐scale defects found in this work. These multi‐scale structural features are highly effective in increasing the phonon scatterings in a wide range of frequency spectrum^[^ ^23^, ^34^ ^]^ so that a very low *κ*_lat_ (Figure 3g) can be realized.

### 2.4 ZT and Conversion Efficiency

**Figure 5:**
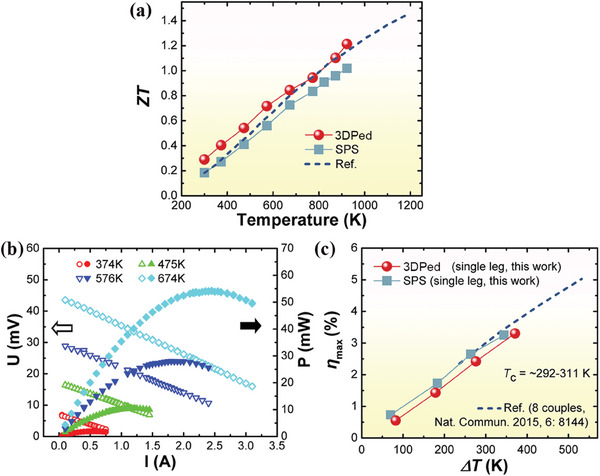
The thermoelectric performance of 3DPed, SPS‐prepared, and the reference.^[^ ^2a^ ^]^ a) *ZT* values as a function of temperature. b) Voltage‐current and power‐current characteristics of the 3DPed single‐leg thermoelectric module measured at a hot‐side set at different temperatures. c) Measured maximum conversion efficiency (*η*_max_) as a function of temperature difference (*ΔT*).

**Figure 6:**
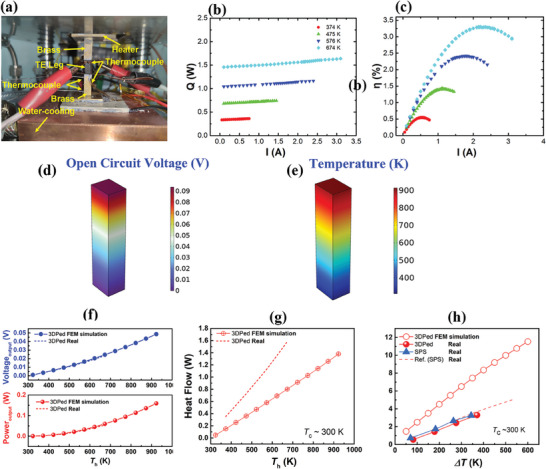
The power generation characteristics of 3DPed sample at different hot‐side temperatures. a) The experimental set‐up of a single‐leg module thermoelectric efficiency testing system. b) Heat flow (*Q*) versus current (*I*). c) conversion efficiency (*η*) versus current (*I*). d,e) Simulated open circuit voltage and temperature distributions in a single 3DPed HH leg. f – h) Comparisons of simulated and measured (f) output voltage and power, g) heat flow, and h) conversion efficiency under the condition of constant 300 K on the cold side. The calculated power generation characteristics are compared with the measured ones.

In **Figure** **5**a, the *ZT* values are provided as a function of temperature. 3DPed possesses a comparable *ZT* value (peak *ZT* = 1.2 @ 923 K) as that of SPSed and ref. [2a] Besides, degradation tests performed after 90 days of atmospheric exposure (Figure S8, Supporting Information) indicate good stability and a similar ultimate *ZT* near room temperature leaving only small deterioration in *σ*. In order to characterize the practical conversion efficiency, a single‐leg thermoelectric module (4.15 × 4.09 mm^2^ in cross‐section and 9.03 mm in height) was made from 3DPed and the current (*I*) dependence of voltage (*U*)/output power (*P*) were tested (Figure 5b). The maximum conversion efficiency (*η*_max_) as a function of temperature difference (*ΔT*) is plotted in Figure 5c, showing the thermoelectric conversion efficiency (*η*_max_) of the LPBF‐prepared sample as ≈3.3% at *ΔT* ≈371 K, which is comparable with that of ref. [2a] (up to ≈674 K on the hot side). It was noticed that the *ZT* value of 3DPed prevailed that of SPS, whereas the measured *η* value was a little bit higher than that of 3DPed. The discrepancy was probably attributed to the different instrumental uncertainties. Nonetheless, the close values in both *η* and *ZT* data reflected the reliability of conversion efficiency results. **Figure** **6**a exhibits a home‐made thermoelectric conversion efficiency testing system for single‐leg module measurement. The cold side is controlled ≈300 K by circulating water and the assistance of a Bi_2_Te_3_‐based cooling plate. The entire system is evacuated to suppress oxidation and heat convection. The corresponding power generation characteristics (heat flow versus current, efficiency versus current) are given in Figure 6b,c. Due to limitation of the testing system, inevitable thermal radiation and over‐estimated *P* and *η*_max_ would be presented if the hot‐side temperature was further increased. Nevertheless, the given *η*_max_‐*ΔT* together with the degradation results (Figure S4, Supporting Information) still reflect the great reliability of the high thermoelectric performance of the 3DPed sample.

The simulated performances of output voltage, output power, heat flow, and theoretical *η* are given in Figure 6d–h based on a finite element method, and the discrepancy between the calculated and measured characteristics mostly results from the considerable amount of heat flow along with the thermal radiation and convection losses in real cases. Moreover, the module/device performances are closely linked with thermoelectric material properties. The relatively smaller *PF* in the 3DPed sample (Figure 3d) probably comes with a low output power. Nonetheless, one should expect efficient heat management and dramatically suppressed heat flow from the hot side to the cold side due to the extremely low thermal conductivity (Figure 3e). Hence, the loss in output power can be compensated and the thermoelectric conversion efficiency of the module‐made from the 3DPed sample is able to catch up with the SPSed and ref. ones. It should be mentioned that the reasonable comparison between a single‐leg module and a p/n couple (dashed line in Figure 5c) is only rationalized for an ideal condition with negligible contact resistance and heat loss. Even so, the *ZT* and single‐leg *η*_max_ in this work are the record‐high performances among 3DP‐prepared HH materials.

### 2.5 Mechanical Property

**Figure 7:**
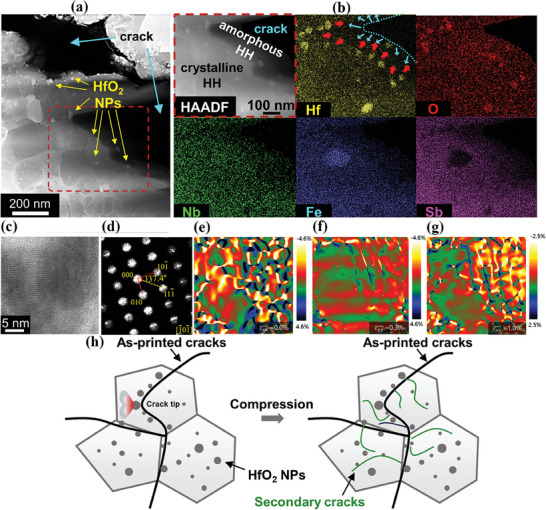
TEM‐related micrographs for a hybrid region in the 3DPed sample. a) HAADF images showing the coexistence of HH matrix, cracks, and HfO_2_ nanoparticles (NPs). b) EDS mappings for hafnium, oxygen, niobium, iron, and antimony elements. The arrows in (b) indicate the confrontation from Hf‐rich NPs against crack propagation. c) An HRTEM image of one Hf‐rich NPs in HH matrix. d) The FFT of (c). e–g) The GPA analyses along *ε*_xx_ (e), *ε*_yy_ (f), and *ε*_xy_ (g) of (c). h) A schematic showing the evolution/initiation of as‐printed/secondary cracks and the energy dissipation under the condition of stress.

Although the LPBF‐based approach is effective for fabricating HH bulks as evidenced by competent *ZT* and *η*_max_‐*T* performance, the non‐fully dense nature of LPBF‐prepared HH samples seems contradictory to the comparable mechanical strength (Figure 2b). We performed a carefully designed destructive test employing the hot isostatic pressing (HIP) method. Applying a pressure of 150 MPa in this procedure exceeded the maximum compressive strength of the printed component (134 MPa), leading to the mechanical failure of the structure. Notably, the hot isostatic pressing process successfully preserved the integrity of the specimen, thereby facilitating the observation of internal mechanical mechanisms. The results revealed that substantial secondary cracks are initiated from the as‐built cracks under stress conditions (Figure S9, Supporting Information). Given the sufficient consumption of fracture energy by the newly generated crack network, the early rupture along as‐built cracks can be effectively prevented. HRTEM was used to further investigate the crack‐initiation and strengthening phenomena in the LPBF‐prepared HH bulks. A hybrid region containing cracks and the adjacent HH matrix is displayed in **Figure** **7**a. A bunch of nanoparticles (NPs) exist along the interfaces between the cracks and the HH matrix. Figure 7b is a high‐angle annular dark field (HAADF) image of an enlarged area framed in Figure 7a. An amorphous HH region, which is a result of over‐bombardment by a focused electron beam, is distinctly recognized between the crystalline HH matrix and the crack. Inside the amorphous region, several crystalline NPs survive, indicating their much higher tolerance against electron irradiation than that of the HH matrix, which is forced to transform into amorphous. According to the EDS elemental analyses (Figure 7b), the solid NPs found along the HH/cracks interfaces contain rich hafnium and oxygen. Further, HRTEM and FFT investigations (Figure 7c,d) identify the NP as monoclinic HfO_2_, which shows a high melting point, high thermal and chemical stability, high hardness, and stable crystal structure. Herein, HfO_2_ NPs served as the strong pinning centers embedded in the HH matrix and effectively hindered the propagation of as‐built cracks (as demonstrated in Figure 7b). Moreover, the HfO_2_ NPs were exclusive to the 3DPed sample as they were absent in the HH LM ingot (Figure S10, Supporting Information). Similar cases were previously reported in NiAl,^[^ ^35^ ^]^ CrMnFeCoNi,^[^ ^36^ ^]^ and 6061Al^[^ ^37^ ^]^ alloys. The geometric phase analyses (GPA) for Figure 7c are given in Figure 7e–g. Stress is observed with centering on HfO_2_ but the overall stress field is negligible due to the offset of local strain. Nevertheless, a relatively large tensile stress (1%) was found along the shear direction (*ε*_xy_), which originates from the lattice distortion of HfO_2_. Hence, excessive stress in HH can be released by the atomic rearrangement in HfO_2_ NPs. Figure 7h systematically elucidates the role of HfO_2_ nanoparticles in impeding crack propagation. Throughout the loading process of the samples, a substantial number of secondary cracks are induced, effectively mitigating excessive local stress concentration and averting premature mechanical failure of the printed components. It again confirms the important role of HfO_2_ pinning centers against crack propagation. These HfO_2_ NPs effectively work as the scattering centers of heat‐carrying phonons. It can be proved in both HfO_2_‐incorporated HH and Bi_2_Te_3_ composites by the evidence of enhanced thermoelectric performances.^[^ ^38^ ^]^

## 3 Conclusion

In summary, we reported a preparation methodology for a high‐quality spherical precursor of Nb_0.88_Hf_0.12_FeSb leading to successful LPBF fabrication. Both high relative mass density and impressive compression strength of 134 MPa were achieved. The potential of LPBF in geometric customization was demonstrated by an arc‐shaped HH design suitable for cylindrical heat sources. EBSD‐TKD observations unambiguously showed the absence of columnar grain and the coexistence of low‐angle‐ and high‐angle‐grain‐boundaries (GBs). They are confirmed to be evidence of FH nano‐inclusions and sub‐GBs, which significantly impact the thermoelectric properties. Although the LPBF‐prepared samples show lower *σ* and *µ* compared to the reference samples due to the non‐fully dense nature and the presence of defects on multiple length scales, they exhibit remarkably suppressed *κ*_lat_ of 1.3 W m^−1^ K^−1^, which compensates for the *PF* degradation. The printed HH material achieved a *ZT* of 1.2 (@ 923 K) and a single‐leg *η*_max_ of 3.3% (Δ*T* = 371 K), which are comparable to the highest efficiencies obtained through conventional manufacturing methods. Furthermore, HfO_2_ nanoparticles were found near the as‐built cracks in the LPBF‐prepared samples, serving as pinning centers to effectively prevent further crack propagation. The integration of the LPBF methodology to fabricate HH described in this study can be extended to the scalable fabrication of other thermoelectric materials, paving the way for a simplified and low‐cost manufacturing process with highly efficient customization capabilities in mid‐to‐high temperature TEGs, such as turbojet engines and radioisotope batteries.

## 4 Experimental Section

### Materials

High purity Nb (99.95%), Hf (99.95%), Fe (99.95%) pellets, and Sb (99.99%) granules were purchased from Zhongnuo Advanced Material (Beijing) Technology Co., Ltd. The raw materials were weighted according to the stoichiometric composition of Nb_0.88_Hf_0.12_FeSb and melted into compound ingots by Levitation melting (LM). Sb (5 wt.% of excess) was added during the melting process to compensate for the mass loss caused by its volatilization. A water‐cooling copper crucible was employed for casting. The feeding sequence of raw materials was determined based on melting points (firstly Hf and Nb, then Fe, and finally Sb), and several intermediate compounds were made. Each time, the base vacuum was below 0.02 Pa and then the chamber was filled with an argon atmosphere of 0.08 Pa. The ingots were remelted 10 times to enhance homogeneity. As for the preparation of spherical HH precursor powder, the Nb_0.88_Hf_0.12_FeSb ingots from LM were transferred into an aluminum oxide crucible placed in a vacuum induction melting inert gas atomization (VIGA) apparatus, and a gas atomization process was conducted by Peshing New Metal (Changzhou) Co., Ltd. The argon intake pressure and temperature were set in the range of 5–7 MPa and 1300–1500 °C, respectively. The base vacuum of the chamber was better than 8 × 10^−3^ Pa, while the argon filling pressure and nozzle diameter were 0.05 MPa and 6–7 mm, respectively. The as‐atomized HH powder was mechanically sieved under 58 µm. The HH spherical powder was dried in an evacuated oven before the LPBF process to remove possible moisture.

### 3D Printing of HH Materials by LPBF

The laser powder bed fusion (LPBF) experiments were performed using an LPBF printing system (HBD SLM‐100, Guangdong Hanbang 3D Technology Co. Ltd.) equipped with a fiber laser (wavelength as 1064 nm) up to 100 W, a powder delivery system, a numerically controlled formation chamber, and a wind field controlling system. The printing parameters were set as follows: laser power 15 W, scanning speed 60 mm s^−1^, hatch distance 50 µm, layer thickness 30 µm. After building five layers in a sequence, the LPBF process was paused for 1 min. An argon atmosphere was used to suppress the oxygen partial pressure under 10 ppm. A post‐annealing heat treatment process at 800 °C for five days was conducted in evacuated quartz tubes (better than 10^−3^ Pa) to improve the chemical homogeneity of LPBF as‐prepared samples.

### Phase, Microstructure, and Mechanical Tests

X‐ray diffraction (XRD) θ−2θ measurements with Cu‐Kα radiation were conducted by an X‐ray diffractometer (DX‐2700, Dandong Haoyuan Co. Ltd.) for examining crystal structure. The surface microstructure of powder and bulk was characterized by a scanning electron microscope (SEM, TESCAN VEGA3). A transmission electron microscope (TEM, Talos F200X G2) was employed for atomic‐scale architecture observations. Lamellae for STEM were prepared through the in situ lift‐out technique in a focused ion beam (FIB, FEI Helios G4CX) system. The particle size distribution of the spherical powder was measured by a laser particle analyzer (Microtrac3500). The flowability of spherical powder was tested by a Hall flowmeter funnel. Five independent pouring measurements (50 g each serve) are performed to estimate an average flow rate. The Hall flow rate of HH spherical powder was evaluated as 13.13 s/50 g, which manifests its excellent flowability exceeding the multiple commercial powders^[^ ^39^ ^]^ for LPBF. Compression examinations of the cuboid samples (3.5 mm × 3.5 mm × 5.0 mm) were performed under uniaxial compression at a constant crosshead speed, corresponding to an initial strain rate of 5 × 10^−5^ s^−1^ at room temperature (25 °C) in a Shimadzu AGX‐VD electronic universal material testing machine with a maximum load capacity of 50 kN. The electron backscatter diffraction transmission Kikuchi diffraction (EBSD‐TKD, detector: HKL Symmetry) was performed on an SEM system (FEI Sicos) to clarify the orientation and grain boundary (GB) properties of nanosized grains. The computed tomography (CT) scanning is conducted in a NanoVoxel‐4000 instrument (Tianjin Sanying) at 150 kV/100 µA. The hot isostatic pressing (HIP) treatment is performed at 880 °C/150 MPa for 60 min in an AIP6‐30H (American Isostatic Presses, Inc.) chamber.

### Thermoelectric Properties Measurements

The testing samples are cut and completely separated from the substrate. The temperature dependence of Seebeck coefficient *S* and electrical conductivity *σ* were measured by a ZEM‐3 apparatus (Advance‐Riko). The total thermal conductivity *κ*_tot_ was calculated by *κ*_tot_ = *DC*_p_ *ρ*, where *D* is the thermal diffusivity collected by a laser flash apparatus (Netzsch LFA 475), *C*_p_ is the specific heat determined based on the law of Dulong‐Petit, and *ρ* is the mass density obtained by the Archimedes method. The empirical measurement uncertainties of *σ*, *S*, and *κ* are ≈4%, 5%, and 7%, respectively. The sample for *σ* and *S* measurements was cut from the one for *κ* measurement. The carrier concentration n was measured using a Physical Property Measurement System (PPMS, Quantum Design). In fabricating the TE single‐leg device, the leg was bridged by copper sheets at the hot side and the cold side using Ag─Cu─Zn solder brazed at 680–730 °C. The output performance of the single‐leg device was measured by a home‐built testing system under a vacuum atmosphere of 10^−6^ mbar. The difference in hot‐side temperatures between the *ZT* (923 K) and the conversion efficiency *η* (674 K) results was attributed to different apparatus for *ZT* and *η* measurements. The finite element method was conducted by using COMSOL 6.0 Multiphysics software to calculate and simulate the heat flow, output power, and conversion efficiency of the single‐leg thermoelectric module. The cold end temperature was maintained at 300 K, while the hot end temperature gradually increased from 300 to 923 K. The calculations performed in the simulation were based on steady‐state analysis. The degradation test was a 90‐day exposure to the atmosphere followed by identical conditions of *S*, *σ*, and *κ* measurements. The single‐leg device was assembled using a soldering process, resulting in dimensions of 2.37 mm × 3.04 mm × 13 mm. The output power and conversion efficiency were tested using a custom‐built test system in a vacuum atmosphere. During the test, one side of the single‐leg device was heated from 300 to 674 K, while the cold side was maintained at room temperature by circulating water. The conversion efficiency is obtained from

(1) $$\eta =\frac{P}{P+Q_{out}}\;\times \;100\%$$

where *P* is the output power of the single‐leg device and *Q*_out_ is the heat flow measured by a flux probe at the cold side.

## Conflict of Interest

The authors declare no conflict of interest.

## Supporting information

Supporting Information

## Data Availability

The data that support the findings of this study are available from the corresponding author upon reasonable request.
