# Supplementary material for: Making High Thermoelectric and Superior Mechanical Performance Nb0.88Hf0.12FeSb Half‐Heusler via Additive Manufacturing
Source: Adv Sci (Weinh). 2024 Sep 9;11(41):2403705. doi: 10.1002/advs.202403705 (PMC11538669; doi:10.1002/advs.202403705)
Supplement: Supplementary file 1 — Supporting Information [file ADVS-11-2403705-s001.pdf]

ADVANCED SCIENCE
Open Access

Supporting Information

for *Adv. Sci.*, DOI 10.1002/advs.202403705

Making High Thermoelectric and Superior Mechanical Performance $Nb_{0.88}Hf_{0.12}FeSb$ Half-Heusler via Additive Manufacturing

Zhifu Yao, Wenbin Qiu, Chen Chen, Xin Bao, Kaiyi Luo, Yong Deng, Wenhua Xue, Xiaofang Li, Qiujun Hu, Junbiao Guo, Lei Yang, Wenyu Hu, Xiaoyi Wang, Xingjun Liu, Qian Zhang*, Katsumi Tanigaki and Jun Tang**

Supplementary Materials for

Making high thermoelectric and superior mechanical performance $Nb_{0.88}Hf_{0.12}FeSb$ half-Heusler via additive manufacturing

Zhifu Yao, Wenbin Qiu, Chen Chen, Xin Bao, Kaiyi Luo, Yong Deng, Wenhua Xue, Xiaofang Li, Qiujun Hu, Junbiao Guo, Lei Yang, Wenyu Hu, Xiaoyi Wan[3], Xingjun Liu, Qian Zhang*, Katsumi Tanigaki, and Jun Tang**

Z. Yao, W. Qiu
Department of Fundamental Courses Wuxi Institute of Technology WuXi 214121, China
E-mail: qiuwb@wxit.edu.cn

Z. Yao, C. Chen, X. Bao, W. Xue, X. Li, X. Liu, Q. Zhang
School of Materials Science and Engineering and Institute of Materials Genome & Big Data Harbin Institute of Technology Shenzhen 518055, China
E-mail: zhangqf@hit.edu.cn

K. Luo, J. Guo, J. Tang
Key Laboratory of Radiation Physics and Technology of Ministry of Education Institute of Nuclear Science and Technology Sichuan University Chengdu 610064, China
E-mail: tangjun@scu.edu.cn

Y. Deng, X. Wang
State Ethnic Affairs Commission Southwest Minzu University Chengdu 610041, China

Q. Hu, J. Tang
College of Physics Sichuan University Chengdu 610064, China

L. Yang
School of Materials Science & Engineering Sichuan University Chengdu 610064, China

W. Hu
Materials Characterization and Preparation Center and Department of Physics Southern University of Science and Technology Shenzhen 518056, China

K. Tanigaki
Division of Quantum State of Matter Beijing Academy of Quantum Information Sciences Beijing 100193, China

* Corresponding author

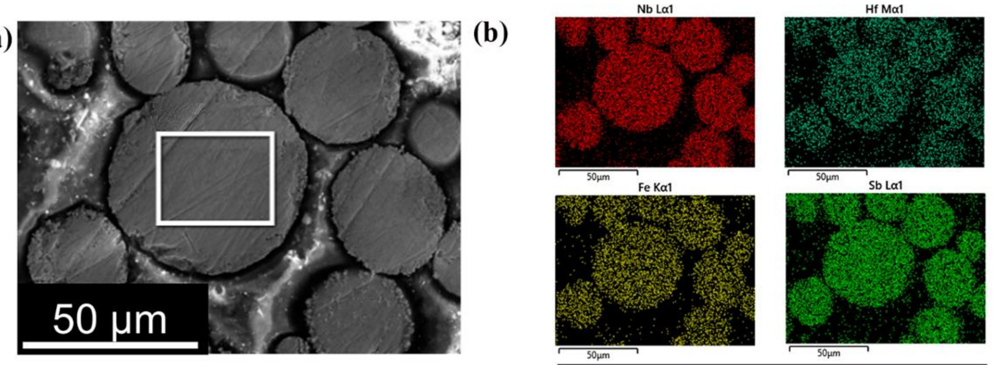

(c)

Element	Signal	at. %
**Nb**	L line	**30.57**
**Hf**	M line	**3.74**
**Fe**	K line	**31.81**
**Sb**	L line	**33.88**
Sum.		100.00

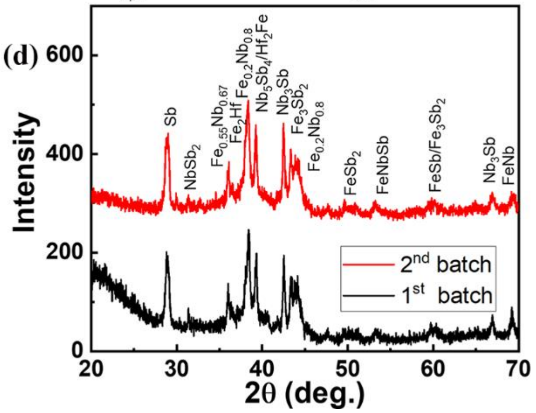

Figure. S1 The morphology, chemical composition, and phase evaluation of the spherical $Nb_{0.88}Hf_{0.12}FeSb$ powder. (a) The cross-sectional SEM image. Most of the powders show a particle size between 13 to 53 μm which is within the optimal window for the subsequent LPBF fabrication. In addition, all powder exhibits dense appearance without any hollow being detected. (b) EDS mappings for niobium, hafnium, iron, and antimony elements. (c) The ratio of the constituent elements in the framed region in (a). (d) XRD 2θ scanning for the spherical powder. Two different batches were both tested.

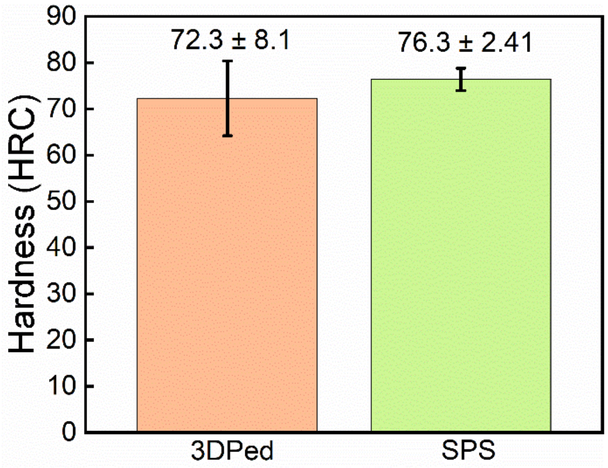

Figure. S2 The hardness of 3DPed and SPS samples.

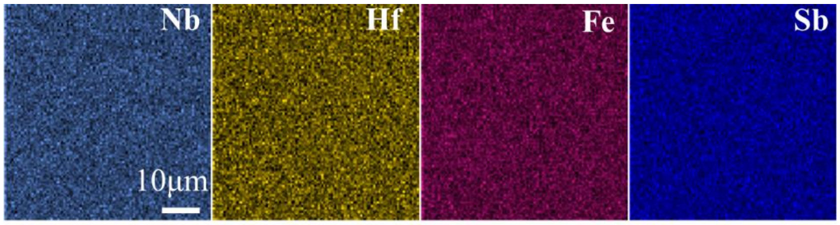

Nb (at. %)	Hf (at. %)	Fe (at. %)	Sb (at. %)
30.1	3.8	33.2	32.9

Figure. S3 EDS-mapping results of 3DPed samples.

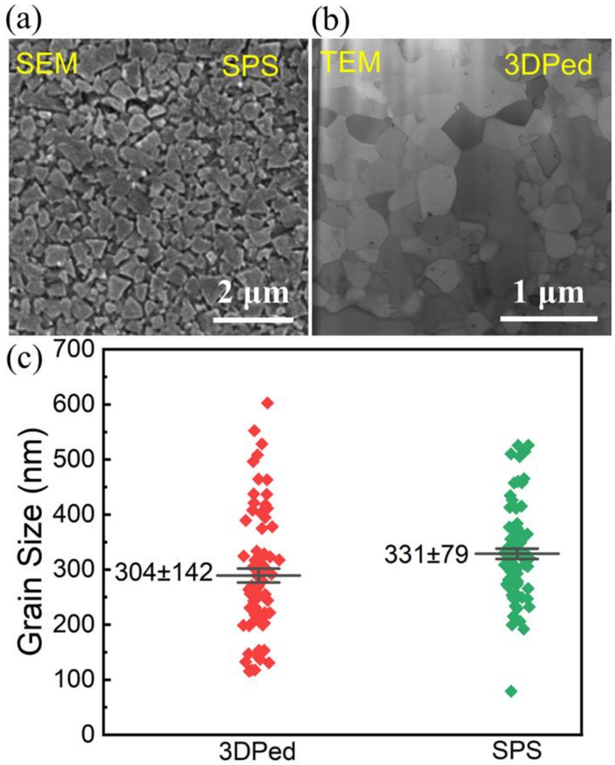

Figure. S4 Microstructure of SPS and 3DPed samples. (a) SEM images of SPS samples; (b) TEM images of SPS samples; (c) Grain size statistics.

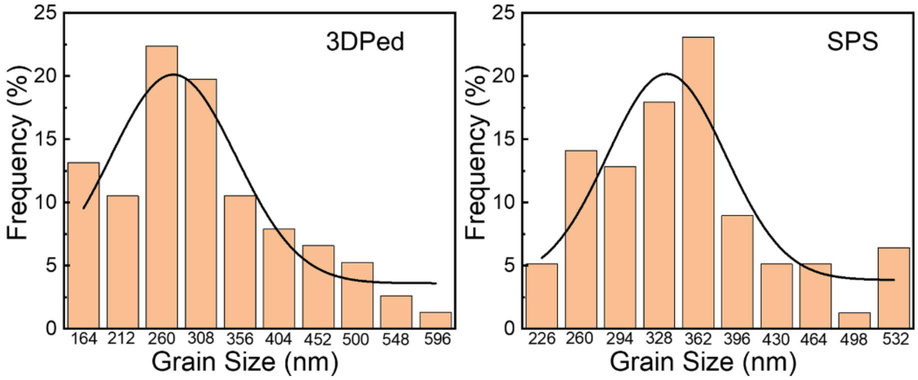

Figure. S5 Grain size distribution statistics.

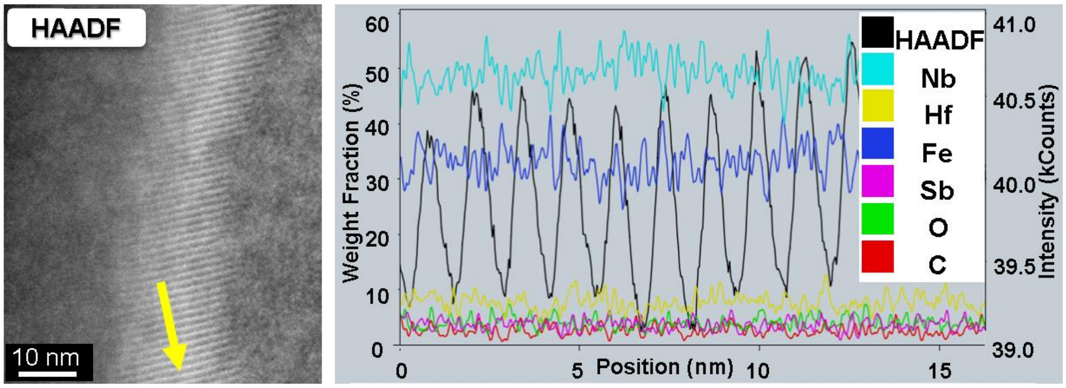

Figure. S6 TEM image (left) and the corresponding EDS linear scanning result (right) for a region with zip-shaped GB in the 3DPed sample. None distinct or periodic fluctuation is observed in any composing elements throughout the scanning path, implying that this defect is induced by edge dislocations. A great amount of zip-shaped GBs are found in 3DPed sample and identified as piling dislocation arrays. Apart from noise signals, the results show almost flat nature for the elemental distribution of Nb, Hf, Fe, Sb, O, and C, verifying the absence of elemental fluctuation or aggregation in these regions by SEM-EDS linear scanning.

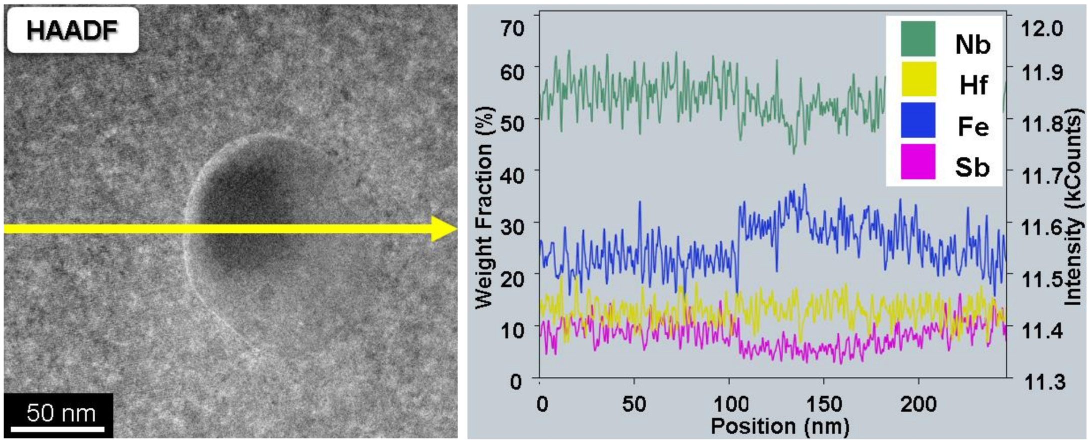

Figure. S7 TEM image (left) and the corresponding EDS linear scanning result (right) for a region with a nano-inclusion in 3DPed sample. The Fe-rich nature in the region of nano-inclusion is clearly noticed, indicating the high possibility of FH phase with doubled amount of Fe sites.

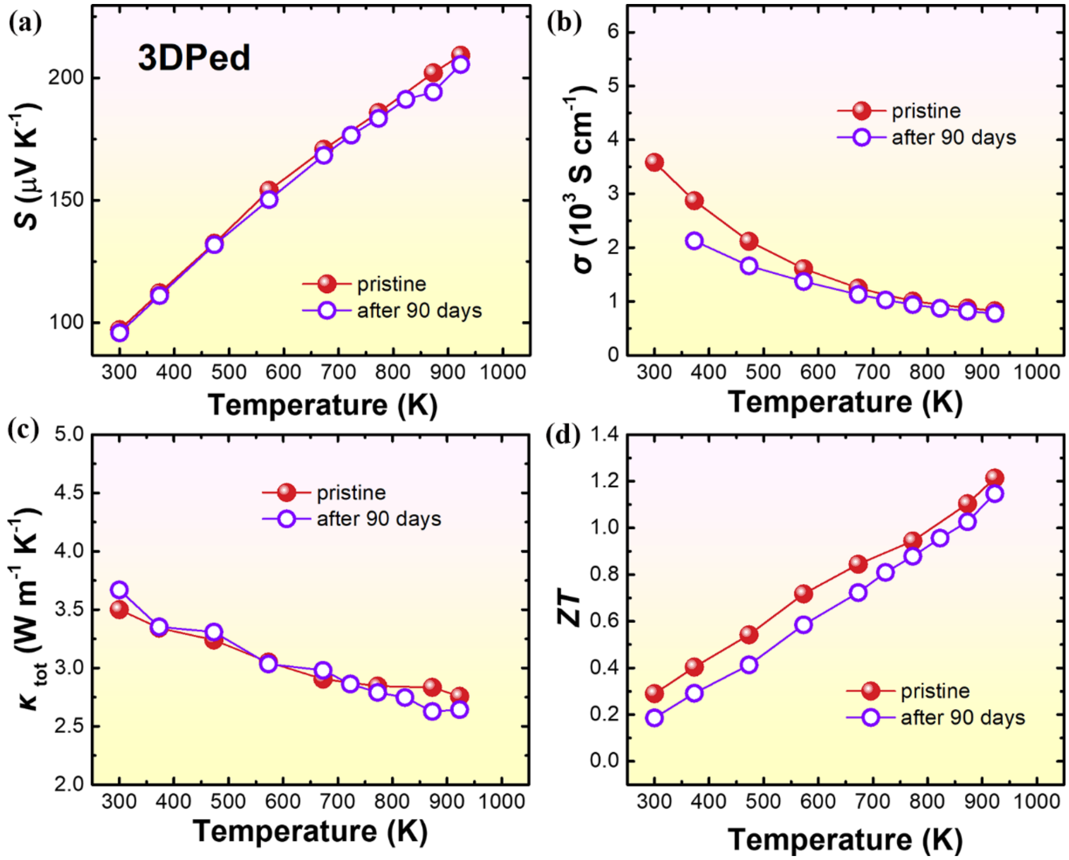

Figure. S8 Degradation tests (90 days exposed in atmospheric environment) for the LPBF-prepared sample. (a) *S*, (b) *σ*, (c) *κ* and (d) the *ZT* of the same 3DPed sample 90 days after the thermoelectric measurements were firstly performed. Almost identical *S* and *κ* values are obtained without degradation. For the case of *σ*, deterioration up to 30% occurs near room temperature after 90 days, which might result from minor oxidation on sample surface. Nevertheless, the performance difference in *σ* becomes negligible at the intermediate-high temperature range within which HH materials are highly operative.

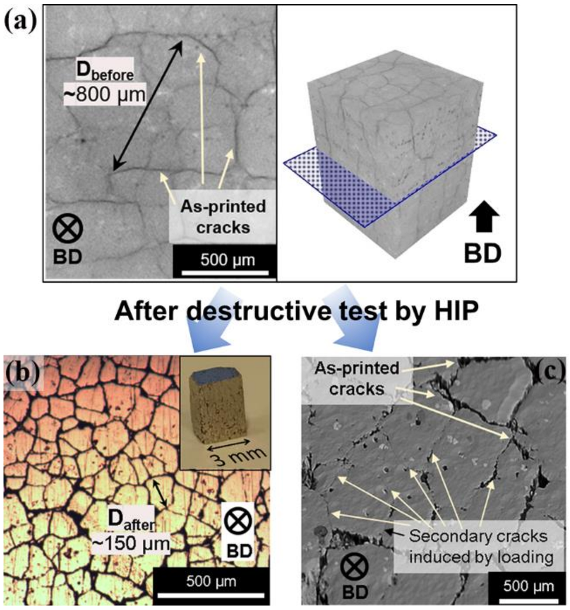

Figure. S9 The crack-related analyses of 3DPed sample. (a) A CT tomographic image for a 3DPed sample from the view along BD. Macroscopic cracks (500-800 μm in distance) are observed throughout the bulk. (b, c) Metallograph and SEM images after a destructive test by a specific hot isostatic pressing (HIP) treatment at 880 °C (service environment of HH-TEG) and 150 MPa (higher than the ultimate compressive strength of 134 MPa). The destructive test of HIP is a perfect way to seize the critical state between the crack initiation and the fracture by fully preserving the sample integrity. The inset of (b) displays the outside appearance of the sample. A great number of secondary cracks (100-200 μm in distance) are observed within the networks of as-printed cracks after HIP loading, revealing extra energy consumption spent in forming fresh cracks rather than exacerbating the as-built cracks in 3DPed under stress.

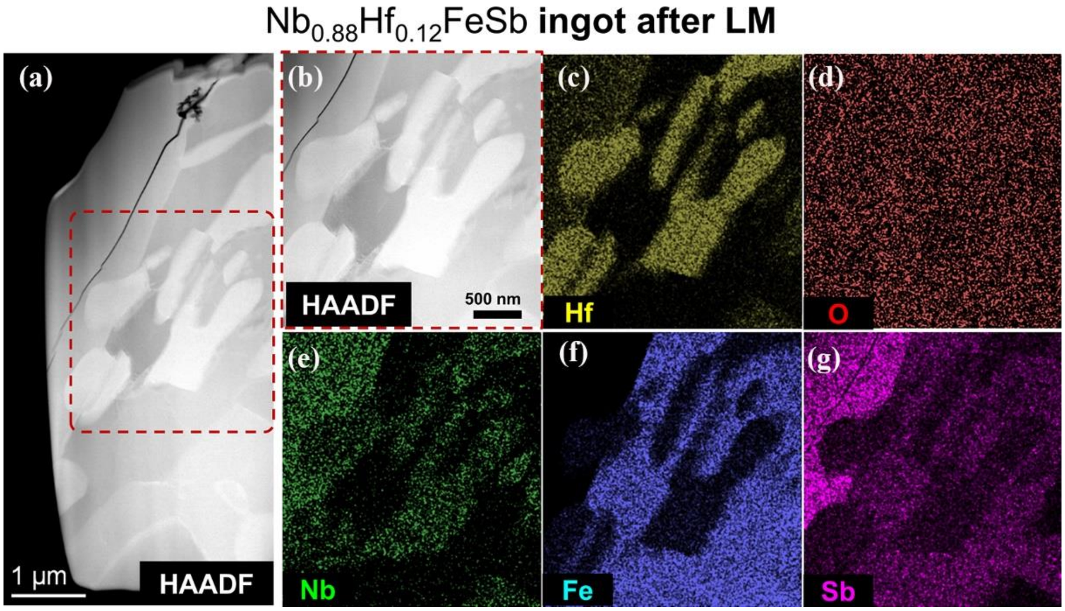

Figure. S10 TEM micrographs for a HH levitation melting (LM) cast ingot. (a, b) HAADF images covering a cracked region. (c-g) EDS mappings for hafnium, oxygen, niobium, iron and antimony elements. Apart from some element segregation of four ingredients, there is no trace of nanosized HfO_2 phase in anywhere of the HH ingot, which excludes LM as a possible origin of HfO_2 nano-particles. Therefore, only the laser powder bed fusion (LPBF) process with extremely high transient temperature on target materials (up to 10^8 K) is able to generate HfO_2 NPs.
